# Cost-Effectiveness Analysis of Option B+ for HIV Prevention and Treatment of Mothers and Children in Malawi

**DOI:** 10.1371/journal.pone.0057778

**Published:** 2013-03-12

**Authors:** Olufunke Fasawe, Carlos Avila, Nathan Shaffer, Erik Schouten, Frank Chimbwandira, David Hoos, Olive Nakakeeto, Paul De Lay

**Affiliations:** 1 Master of International Health Management, Economics and Policy Program, SDA Bocconi School of Management, Milan, Italy; 2 Senior Health Economist, Principal Associate, Abt Associates, Bethesda, Maryland, United States of America; 3 PMTCT Technical Lead, HIV Department, World Health Organization, Geneva, Switzerland; 4 HIV Advisor, Management Sciences for Health, Lilongwe, Malawi; 5 Director of the HIV and AIDS Department, Ministry of Health, Lilongwe, Malawi; 6 Assistant Professor of Clinical Epidemiology, Senior Implementation Director, ICAP, Columbia University, Mailman School of Public Health, New York, New York, United States of America; 7 Health Economist, Independent Consultant, Saint-Genis-Poully, France; 8 Deputy Executive Director, Joint United Nations Programme on HIV/AIDS (UNAIDS), Geneva, Switzerland; Indiana University and Moi University, United States of America

## Abstract

**Background:**

The Ministry of Health in Malawi is implementing a pragmatic and innovative approach for the management of all HIV-infected pregnant women, termed Option B+, which consists of providing life-long antiretroviral treatment, regardless of their CD4 count or clinical stage. Our objective was to determine if Option B+ represents a cost-effective option.

**Methods:**

A decision model simulates the disease progression of a cohort of HIV-infected pregnant women receiving prophylaxis and antiretroviral therapy, and estimates the number of paediatric infections averted and maternal life years gained over a ten-year time horizon. We assess the cost-effectiveness from the Ministry of Health perspective while taking into account the practical realities of implementing ART services in Malawi.

**Results:**

If implemented as recommended by the World Health Organization, options A, B and B+ are equivalent in preventing new infant infections, yielding cost effectiveness ratios between US$ 37 and US$ 69 per disability adjusted life year averted in children. However, when the three options are compared to the current practice, the provision of antiretroviral therapy to all mothers (Option B+) not only prevents infant infections, but also improves the ten-year survival in mothers more than four-fold. This translates into saving more than 250,000 maternal life years, as compared to mothers receiving only Option A or B, with savings of 153,000 and 172,000 life years respectively. Option B+ also yields favourable incremental cost effectiveness ratios (ICER) of US$ 455 per life year gained over the current practice.

**Conclusion:**

In Malawi, Option B+ represents a favorable policy option from a cost-effectiveness perspective to prevent future infant infections, save mothers' lives and reduce orphanhood. Although Option B+ would require more financial resources initially, it would save societal resources in the long-term and represents a strategic option to simplify and integrate HIV services into maternal, newborn and child health programmes.

## Introduction

HIV continues to pose a serious health risk for pregnant women and their children in high prevalence settings. Vertical transmission, occurring during pregnancy, labour, delivery or breastfeeding [1], remains the main mode of HIV infection in children. An estimated 390 000 children globally acquired HIV from their mothers in 2010 with over 90% of these new infections occurring in sub-Saharan Africa [2]. While the majority of infants of HIV-infected mothers do not themselves become HIV-infected, they are nonetheless at risk of increased mortality and morbidity and vulnerable to orphanhood [3]. However, the use of antiretroviral drugs during and after pregnancy is a proven intervention to virtually eliminate the risk of HIV transmission to infants, as evidenced in high-income countries where new childhood HIV infections are now almost non-existent [4], [5].

Malawi, a low-income country of 15 million people is one of the countries with the highest number of HIV-infected pregnant women; between 57,000 and 76,000 pregnant women (mid-point estimate 66,500) were HIV-infected and required antiretroviral prophylaxis for prevention of mother-to-child transmission (PMTCT) in 2010 [2]. There are approximately 663,000 annual births and a high mortality ratio (510/100,000 births); approximately 32% of maternal deaths are attributable to HIV [6].

Malawi has experienced successful national efforts in reducing disparities in safe motherhood with reductions in maternal mortality of approximately 50% in the last decade. More than 90% of pregnant women attend antenatal clinics at least once during their pregnancy [7], although the majority first attend during the second or third trimester. The Government of Malawi has implemented a decentralized approach to HIV prevention, care and treatment in order to reach the 85% of Malawi's population that live in rural areas [8]. Malawi has also had notable success in rapidly expanding ART (antiretroviral treatment) coverage in the general population; the number of ART sites across the country grew from 9 to 491 between 2003 and 2009, almost half of which are community-based health centres, and an estimated 49–57% of HIV-infected adults eligible by clinical or immunologic criteria were receiving ART by the end of 2010. By contrast, the coverage of antiretroviral prophylaxis for HIV-infected pregnant women was still very low in 2010, within the range of 23–31% [2]. Malawi's healthcare system remains overstretched, with one doctor for every 49 000 people and one nurse for every 1 800 people [7] which is ten times lower than the World Health Organization (WHO) recommended minimum standard.

The revised 2010 WHO guidelines for prevention of mother-to-child transmission of HIV recommend lifelong ART for women with CD4 counts at or lower than 350 cells/ µL. The guidelines recommend two prophylaxis regimens for women who are not clinically or immunologically eligible for ART [9]. Option A consists of antepartum zidovudine (AZT) from 14 weeks of pregnancy, single-dose nevirapine (sd-NVP) at the onset of labour and a dual-drug regimen of zidovudine (AZT) and lamivudine (3TC) until one week after delivery. The infant receives daily oral nevirapine from birth until all breastfeeding has ceased. In Option B, mothers receive triple-drug antiretroviral prophylaxis starting from 14 weeks of pregnancy until all exposure to breast milk has ended. Daily oral nevirapine to the infant is provided from birth until six weeks of age. Determination of which women are eligible for lifelong ART and which women receive prophylaxis is primarily through CD4 screening.

The Ministry of Health in Malawi proposed and has recently begun implementing a new approach termed Option B+ in which all pregnant women who test HIV positive are placed on ART for life, from 14 weeks gestation or first antenatal visit, and regardless of their CD4 count or clinical stage [10]. This simplified approach would facilitate the achievement of not only the Global Plan target of elimination of new paediatric HIV infections by 2015 [11], but also the target of universal access to HIV treatment for mothers in a setting where it is difficult to effectively distinguish between those mothers eligible for treatment and those needing prophylaxis. While CD4 testing should be available to guide the initiation of ART [12], Malawi, like many other low-income countries, suffers major constraints in the expansion of laboratory capacity, and specifically regarding access to CD4 testing [13]. The simplification of drug regimen options may also help to improve adherence to therapy and reduce the many bottlenecks within the cascade of PMTCT interventions as countries adopt the Treatment 2.0 framework of simplified HIV treatment [14]. Implementing Option B+ may be a more effective PMTCT strategy, as it can help overcome some of the individual, organizational and societal barriers associated with achieving high coverage levels of prophylaxis and treatment, and will ensure that most HIV-infected pregnant women are placed on treatment immediately following diagnosis leading to further reduction of MTCT [10].

The objective of this analysis was to determine if Option B+ represents a cost-effective policy option for the treatment of HIV-infected pregnant women and for PMTCT, as compared with WHO Options A and B. We assess the cost-effectiveness from the Ministry of Health perspective while taking into account the practical realities and costs of implementing ART services in Malawi.

## Methods

### Model Structure

**Figure 1:**
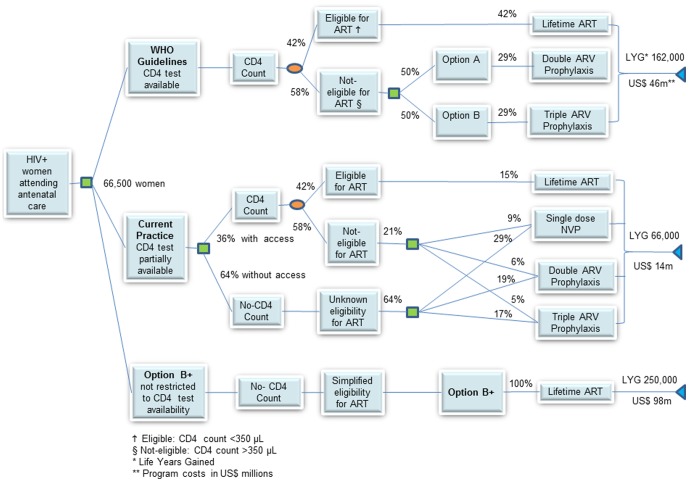
Abbreviated decision tree summarizing the analytical approach, policy options and results.

A decision analytic model was developed to compare the costs, health outcomes and cost-effectiveness of WHO PMTCT Options A and B as well as Option B+ in Malawi for all HIV-infected pregnant women. The analysis was structured as a probability tree starting with an HIV-infected pregnant woman entering into contact with the health system for antenatal care, then receiving a cascade of interventions towards reducing the risk of transmission to her infant as well as care and treatment for her own health (Figure 1). The risk of transmission depends on the background HIV transmission rates during pregnancy, labour, delivery and breastfeeding, the efficacy of the antiretroviral drugs in preventing transmission, prophylaxis and treatment coverage and the level of adherence. The estimated number of HIV-infected pregnant women in Malawi was taken as the midpoint of the range reported for the end of 2010 [2] and this number, 66,500, was then used as an input to analyse as an annual cohort of HIV-infected pregnant women that, after surviving childbirth, was followed over a 10-year time horizon. This time horizon was thought to be sufficiently long enough to capture the effects of immediate or later access to treatment and to assess maternal survival outcomes.

The patterns of disease progression in HIV-infected pregnant women with and without ART were assumed to be similar to that documented in the general adult population. A Markov model was developed to simulate the natural history of HIV infection and project the 10-year maternal outcomes associated with the different options implemented. The model assumes that women are treatment naïve and in one of four transition states: (a) CD4 counts >350 cells/ µL; (b) CD4 counts 350–200 cells/ µL; (c) CD4 between 199–0 cells/ µL and (d) death, as the absorbing state. Women starting with high CD4 counts and subsequently becoming eligible for ART when their CD4 cell counts fall to 350 or below, would access ART services when they move into the eligible transition state, while women eligible for treatment receive life-long therapy and are accounted for in the PMTCT programme. As more women access ART services in the future, the background survival rates of HIV-infected mothers under Options A and B improve when compared to the no-treatment scenario.

Outcomes assessed include the number of infant infections averted, cost per infection averted, cost per disability adjusted life year (DALY) averted for infants, life years gained in HIV-infected mothers, cost per life year gained for the HIV-infected mothers and the incremental cost-effectiveness ratios for each outcome. DALYs were estimated using standard published methods [15].

### Strategies being compared

We analyzed four strategies including; (1) Current practice in 2010, (2) Option A, (3) Option B, and (4) Option B+. For the current practice, we modelled the mix of interventions in Malawi including HIV testing and counselling and ARV prophylaxis for HIV-infected pregnant women at the reported coverage levels as of the end of 2010 [2].We modelled an ante-natal care (ANC) coverage of 91% according to the World Health Statistics report [7] with an HIV testing and counselling coverage of 66% in pregnant women [8]. Using data derived from a direct report from the Ministry of Health (MOH) in Malawi, of the 45% of HIV-infected pregnant women who received antiretrovirals for PMTCT in 2010, 38% received single-dose nevirapine (NVP), 25% received a dual-drug regimen containing zidovudine (AZT), 22% received triple-drug ARV prophylaxis and 15% received full ART [16]. These coverage rates were modelled as the current practice in 2010, prior to the start of the B+ programme. The options analysed and their components are shown in Table 1. In Option A, pregnant women not eligible for ART based on the disease stage were modelled to receive zidovudine from 14 weeks, a single dose of nevirapine at the onset of labour and a dual-drug regimen of zidovudine and lamivudine (AZT+3TC) for one week after delivery. In Option B, pregnant women not eligible for ART were modelled to receive triple-antiretroviral prophylaxis of tenofovir, lamivudine and efavirenz (TDF+3TC+EFV: this is the regimen adopted by Malawi) from 14 weeks until one week after all breastfeeding has stopped. In Option B+, treatment decisions are not based on CD4 count and therefore all HIV-infected pregnant women who receive HIV testing and are positive based on the prevalence rate assumptions, were modelled to initiate and continue lifelong ART. Infants receive prophylaxis with nevirapine until one week after all breastfeeding has stopped in Option A, while they receive nevirapine for six weeks after birth with current practice, Options B and B+.

**Table:** **Table 1.** ARV regimens for HIV prevention and treatment of mothers and children compared in the analysis - Current Practice 2010, WHO Option A, WHO Option B and Malawi's Option B+.

ARV regimens for mothers who do not need treatment for their own health			All HIV-infected mothers
Current Practice 2010	Option A	Option B	Option B+
**Mother**	**Mother**	**Mother**	**Mother**
Depending on availability and setting, single-dose nevirapine (NVP), or dual-drug regimen containing zidovudine (AZT), or triple-drug ARV prophylaxis until cessation of breastfeeding	Antepartum twice-daily AZT starting from as early as 14 weeks of gestation and continued during pregnancy. At onset of labour, sd-NVP and initiation of twice daily AZT+3TC for 7 days postpartum (Note: If maternal AZT was provided for more than 4 weeks antenatally, omission of the sd-NVP and AZT+3TC tail can be considered; in this case, continue maternal AZT during labour and stop at delivery).	Triple ARV prophylaxis starting from as early as 14 weeks of gestation and continued until delivery, or, if breastfeeding, continued until 1 week after all infant exposure to breast milk has ended. Regimen: TDF+3TC+EFV	Antiretroviral therapy starting from as early as 14 weeks of gestation and continued for life. Preferred regimen: TDF+3TC+EFV
**Infant**	**Infant**	**Infant**	**Infant**
**Irrespective of mode of infant feeding:** Daily NVP or twice daily AZT from birth until 4 to 6 weeks of age.	For breastfeeding infants: Daily NVP from birth for a minimum of 4 to 6 weeks, and until 1 week after all exposure to breast milk has ended. **Infants receiving replacement feeding only:** Daily NVP or sd-NVP+twice−daily AZT from birth until 4 to 6 weeks of age.	**Irrespective of mode of infant feeding:** Daily NVP or twice daily AZT from birth until 4 to 6 weeks of age.	**Irrespective of mode of infant feeding:** Daily NVP or twice daily AZT from birth until 4 to 6 weeks of age.

For all strategies, it was assumed that 42% of HIV-infected pregnant women have CD4 counts at or less than 350 cells/ µL and are eligible for ART, based on findings of a population-based study in Malawi [13]. Therefore women eligible for ART under WHO Options A and B receive ART initially in the model and women who later become eligible after delivery and breastfeeding will start to receive ART according to their disease progression and at the coverage of ART in the general population [9]. For this analysis, full ANC coverage was assumed, and all pregnant women were assumed to receive HIV testing and counselling while ARV coverage rates for prophylaxis and treatment were assumed to be 90% for the three options while the 2010 coverage levels reported by the MOH were modelled for the current practice.

### Input parameters

All the input parameters are presented in Table 2. For perinatal transmission rates, we used the recently revised mother-to-child transmission rates by the UNAIDS epidemiology reference group for use in Spectrum [17] along with published findings from the multicentre Kesho Bora study with rates of 2.7% for Option A and 1.7% for Options B and B+ for the postnatal transmission in all options [18]. Breastfeeding can contribute as much as 42% to the overall mother-to-child transmission [19]. The 2010 Demographic Health Survey for Malawi reports a mean breastfeeding duration of 23 months among all mothers [20] and study findings from Malawi report a median breastfeeding duration of 11.5 months among HIV-infected women. [21]. In this analysis, we assumed that HIV-infected mothers would breastfeed for an average of 12 months. The monthly risk of postnatal HIV transmission through breastfeeding, in the absence of any intervention was assumed to be 1.04% which is the average of the rates reported for transmission in eligible and non-eligible women by the UNAIDS epidemiology reference group [18].

**Table 2:** Input parameters and plausible ranges used for sensitivity analysis and relevant references for the Malawi analysis (US $ 2010).

Parameters	Base-case	References
**HIV Epidemiology**		
1. Number of HIV-infected pregnant women	66,500 (57,000–76,000)	[2]
2. Percentage of pregnant women with CD4 count >350 cells/ µL (%)	58	[13]
3. Percentage of pregnant women with CD4 count 349–200 cells/ µL (%)	22	Same as above
4. Percentage of pregnant women with CD4 count <200 cells/ µL (%)	20	Same as above
**MTCT transmission rates**		
5. Background transmission rate without intervention (peripartum)%	22	[17]
6. Monthly post-natal transmission, no prophylaxis, breastfeeding (12 months)%	1.04	[17]
7. Peripartum transmission, Option A %	2.7	[17]
8. Monthly post-natal transmission with infant prophylaxis, breastfeeding (as per Option A)%	0.2	[17]
9. Peripartum transmission, Option B, Option B+ and eligible women on ART %	1.7	[17]
10. Monthly postnatal transmission with ART, breastfeeding (Options B and B+ and women on ART)%	0.2	[17]
**Costs**		
11. HIV Testing and counselling	$3.50	MOH Malawi
12. CD4 Screening	$20.00	MOH Malawi
13. Follow-up visit/clinical monitoring (per visit)	$2.00	MOH Malawi
14. Single-dose NVP	$0.20	MOH Malawi
15. AZT (6 months) and AZT+3TC (7 days)	$60	MOH Malawi
16. TDF+3TC+EFV (per year)	$193.6	MOH Malawi
17. Infant NVP including syringes (per year)	$16.00	MOH Malawi
18. Early infant diagnosis	$32.50	[35]
19. Cotrimoxazole prophylaxis (per year)	$5.00	[36], [37]
21. Discounted lifetime cost for an HIV infected child on ART	$ 3195	[39], [40]

Survival estimates from the model were calibrated with results from the ART-LINC, which reported data from 36,615 patients on ART from 17 cohorts in Africa, Asia and South America [22]. Transition probabilities between adjacent states for untreated women were assumed to be similar to reported findings in other populations and were estimated from the published natural history studies [23]–[28] and calculated as the reciprocals of the mean waiting times in each CD4 cell state [29]. For women receiving ART, the transition probabilities from one state to the next lower one were calculated by applying the reported relative risk of disease progression with treatment compared to no treatment of 0.27 [30]. Weighted averages from studies reporting ART survival in low-income settings were used to determine the mortality rate in each state [31]. Women receiving ART could also progress from lower to higher states based on study findings on CD4 cell recovery for people on treatment [32], but these women are assumed to remain on treatment. The model incorporates possible treatment failures, which can result from poor adherence and loss to follow up (LTFU) during treatment. For the purpose of this analysis, an adherence rate of 90% was used, as reported adherence rates in resource-limited settings are comparable to those in developed countries [33]–[34].

### Cost Estimates

The cost per patient-year in each Markov state was calculated by multiplying health care utilization by the cost per person per year, based on recent cost estimates in Malawi. All costs are shown in US dollars. The cost of rapid HIV testing was $3.50 per test and all pregnant women are assumed to undergo at least one HIV test during the first antenatal visit for Options A, B and B+ while the coverage rates for ANC and HIV testing and counselling were applied for the current practice. HIV-infected pregnant women undergo CD4 cell count testing with the Current Practice, Options A and B to determine eligibility for ART at a cost of US$ 20 per test. Costs of ARVs for Options A and B included 6 months during pregnancy and 12 months during breastfeeding. The cost of drugs per woman receiving Option A for 6 months of prophylaxis is US$ 76.20, consisting of $60 for AZT and AZT+3TC, $0.20 for NVP and $16 for infant Nevirapine for 12 months of breastfeeding. The annual cost of drugs per woman receiving Option B or Option B+ is US$ 193.60 per woman. Based on the percentage distribution of regimens under the current practice as previously described, the same costs of the various regimens were applied to the current practice. Total costs of ARV prophylaxis and ART for all options are calculated for the entire period of 10 years.

Other itemized costs for all the options and the current practice include early infant diagnosis testing, and cotrimoxazole prophylaxis for HIV-exposed infants. Ninety percent of all infants born to an HIV-infected mother are assumed to be tested with DNA PCR at a cost of US$ 32.50 [35]. Cotrimoxazole prophylaxis was assumed to be given to all HIV-exposed infants for a period of 12 months at a cost of US$5 per person per year to account for the time it takes to receive early-infant diagnosis (EID) results and place HIV-infected infants on treatment [36]–[37].

Costs are shown in Table 3. Cost estimates and prices were obtained directly from the Ministry of Health of Malawi, and supplemented with additional cost data from the World Health Organization Global Price Reporting Mechanism [38]. All costs are expressed in US dollars at a 2010 price base. Costs and benefits were discounted at 3% annually. The three options were compared to the current practice using incremental cost-effectiveness ratios, of US dollars per life-year gained and per disability-adjusted life-year averted.

**Table 3:** Costs and paediatric outcomes from preventing mother to child transmission programmatic interventions for 18 months of prophylaxis and treatment* (US $ 2010).

	Current Practice	Option A	Option B	Option B+
**Programmatic Activity**				
HIV testing and counseling	$ 139,789.7	$ 232,750	$ 232,750	$ 232,750
CD4 Testing	$ 455,314.9	$ 1,197,000	$ 1,197,000	$ 0**
Cost of ARVs for prophylaxis and treatment (including monitoring)	$ 2,984,,445.2	$ 8,860,309.6	$ 17,725,341.8	$ 17,725,341.8
Infant prophylaxis	$ 39,523.4	$ 844,603.2	$ 97,454.2	$ 97,454.2
Early infant diagnosis	$ 0.0	$ 1,906,222.5	$ 1,906,222.5	$ 1,906,222.5
Cotrimoxazole prophylaxis	$ 53,521.2	$ 131,969.3	$ 131,969.3	$ 131,969.3
Total PMTCT programme cost (18 months)	$ 3,672,594.3	$ 13,172,854.6	$ 21,290,737.8	$ 20,093,737.5
**Pediatric outcomes**				
Number of infants infected***	16,179	5,075	4,684	4,684
Number of infections averted	4,503	15,606	15,997	15,997
Lifetime costs of averted ART and hospital care among children	$ 14,385,762	$ 49,861,725	$ 51,110,042	$ 51,110,042
DALYS averted	101,308	351,139	359,930	359,930
**Cost-effectiveness ratios**				
Cost per infection averted	$ 816	$ 844	$ 1,331	$ 1,265
Cost per DALY averted	$ 37	$ 37	$ 60	$ 57
ICER per DALY (compared to the current practice)		$ 38	$ 68	$ 64

* Assumes 663,000 pregnant women, 66,500 HIV-infected pregnant women annually, and 90% (59,850) of those women reached by Option A, B and B+.

** Assumes no needed CD4 to start ART under the Malawi Option B+ approach; however, in practice some HIV-infected pregnant women will have access to CD4 testing as part of staging and response to treatment

*** Background infections if no ARV interventions = 20,681

Sensitivity analysis was performed to assess the robustness of the results to changes in the assumptions made. The efficacies of ARV prophylaxis and ART on reducing peripartum and postnatal transmission rates were varied using ranges reported in the literature to take into account clinical practice situations where adherence may be lower than reported in clinical trials.

## Results

Using the current PMTCT practice and coverage in Malawi in 2010 as our base case, a cohort of HIV-infected pregnant women would result in an estimated 16,217 infant infections (24.4% transmission rate) after one year and an estimated 28% survival among HIV-infected mothers after ten years. Our counterfactual analysis showed that without any PMTCT interventions, an estimated 20,681 infections (31.1% transmission rate) would occur after 12 months of breastfeeding. In addition, if no antiretroviral treatment is provided to HIV-infected mothers, the natural HIV progression results in a 3% survival rate after 10 years or an estimated 1,920 women surviving from the initial cohort of HIV-infected women.

### Cost-effectiveness analyses: preventing new paediatric infections

Table 3 shows the costs, outcomes and cost-effectiveness of different strategies modelled to prevent new child infections in the HIV-infected pregnant women. Our base case represents the current PMTCT practice and coverage in Malawi in 2010; at reported coverage levels, this resulted in 4,503 infections averted among infants, with a generalized cost effectiveness ratio of US$ 816 per infection averted.

If fully implemented, Option A averts 391 infections fewer than Options B and B+ and has a generalised cost-effectiveness ratio of $ 844 per infection averted; Option B, a generalised cost-effectiveness ratio of $1,331 per infection averted and Option B+ a generalised cost-effectiveness ratio of US$ 1,265 (from savings attributable to not carrying out CD4 testing). Generalized cost-effectiveness analysis for the three approaches resulted in costs per DALY of US$ 37 for Option A, US$ 60 for Option B and US$ 57 for Option B+.

The incremental cost effectiveness per DALY when compared to the current practice as the base case resulted in ratios of US$ 38 for Option A, US$ 68 for Option B and US$ 64 for Option B+. With comparable coverage and implementation, Option A emerges as the most cost-effective option to prevent new child infections among HIV-infected pregnant women following delivery and 12 months of breastfeeding.

### Cost-effectiveness analysis: maternal health outcomes (10-year analysis of cohort of HIV-pregnant women)

Table 4 presents the assessment of the different options in terms of costs and maternal outcomes for the original annual cohort of HIV-infected pregnant women followed over a ten-year horizon. The total discounted cost of the current practice (limited coverage) amounts to US$ $14.3 million, which is a fraction of the most costly Option B+, at US$ 97.7 million. The current practice in 2010 results in a survival rate of 27.5%, with 18,267 of 66,500 HIV-infected mothers remaining alive after ten years and 66,289 life-years saved. Options A and B result in 152,966 and 171,543 life-years saved respectively. However, Option B+ improves the survival and results in 249,576 life-years saved. The ICER per life-year gained, when each option is compared to the current practice, is US$ 314 for Option A, US$ 338 for Option B and US$ 455 for Option B+.

**Table 4:** Costs, maternal health outcomes and cost-effectiveness ratios of options A and B and Malawi's Option B+ for the ten-year horizon (US $ 2010).

	Current Practice	Option A	Option B	Option B+
**Costs**				
PMTCT costs (first 18 months)	$ 3,672,594	$ 13,172,855	$ 21,290,738	$ 20,093,738
Cost of ART for eligible women (subsequent years)	$ 10,162,136	$ 26,639,260	$ 26,837,855	$ 73,852,060
Cost of follow up and monitoring	$ 448,525	$ 1,692,548	$ 1,692,548	$ 3,784,190
Total programme costs (10 years)	$ 14,283,255	$ 41,504,663	$ 49,821,141	$ 97,729,988
**Outcomes**				
Number of HIV infected women on ART and alive after ten years	18,267	28,567	30,057	42,137
Life years gained in HIV infected mothers after ten years	66,289	152,966	171,543	249,576
**Cost-Effectiveness Ratios**				
ICER per life year gained (compared to the current practice)	-	$ 314	$ 338	$ 455

**Figure 2:**
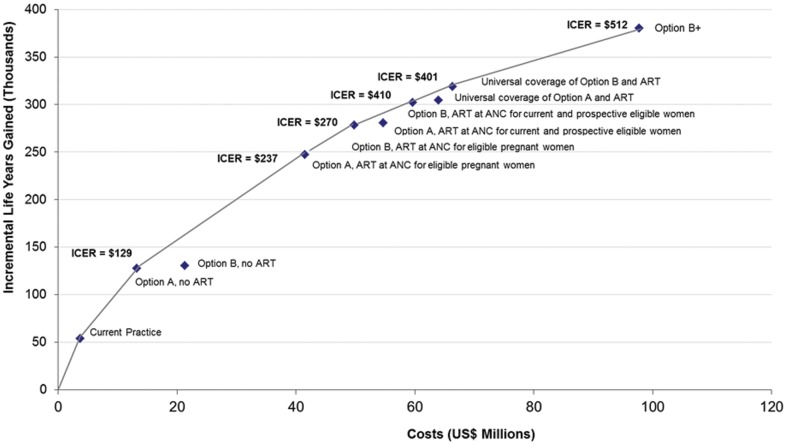
Cost effectiveness of various strategies for the prevention of new pediatric infections and the treatment of HIV-infected mothers in Malawi. Current practice represents our base case scenario or the status quo in 2010. The next set of scenarios highlight the cost effectiveness of incrementally expanding program implementation and service delivery coverage, and ranges from PMTCT only to the addition of integrated ART-ANC services for eligible pregnant women, both identified immediately and at a later time. Universal coverage implies the availability of HIV services for mother and children at any point of needing treatment. Option B+ offers ART to pregnant women regardless of CD4 count.

We evaluated a range of possible scenarios with increasing levels of service coverage for ARV prophylaxis and ART with the different Options and estimated the incremental cost-effectiveness among next best alternative strategies for Malawi. Figure 2 presents the relationship between level of investment and the effectiveness of the intervention expressed in Life Years Gained (LFG). The upward-sloped line represents the boundary and it is called the ‘efficient frontier’. The efficient frontier offers the highest expected return for a given level of investment. The ICERs shown represent the incremental ratios from one alternative to the next best alternative outlined at the frontier line. Current practice represents our base case scenario or the status quo in 2010 and results in the lowest health gains. In terms of outcomes; Option A reaches the efficiency frontier by requiring fewer resources than option B and producing almost comparable outcomes. Full implementation of Option A coupled with the provision of ART to all eligible women results in rapid health gains, with 250 thousand life years over an investment of US$ 50 million. Doubling the investments to US$ 100 million, by providing full Option B+, results in an increase of 120 thousand life years gained.

### Sensitivity analysis

Sensitivity analysis was performed to assess the robustness of the results. For infant outcomes, Option A remained the most cost-effective option with changes in the efficacy of antiretroviral drugs, changes in transmission rates and costs of antiretroviral drugs. The cost of ARVs was varied by 50% below and above the reported price. One-way sensitivity analyses were performed on key model parameters with the model being robust to most of the changes.

**Figure 3:**
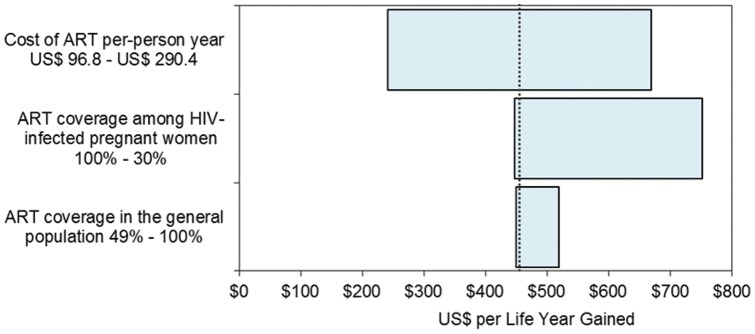
Tornado diagram for the ICER of Option B+, base case is $455 per life year gained shown with the dotted line.

A break-even point between Option B+ and Option A occurs when the annual cost of antiretroviral treatment and care is reduced and reaches $387, with Option B+ becoming the more cost-effective option to prevent new infant infections and producing an ICER of $52.4 per DALY averted in infants. However, when considering outcomes for HIV- infected mothers, the model is sensitive to changes in the coverage of CD4 testing, ARV coverage among HIV-infected pregnant women, ART coverage in the general population and the cost of ART. Results are shown in Table 5. As the coverage of ART in the general population increases, the cost-effectiveness ratio of Option B+ increases while Options A and B become more cost-effective. Option B becomes the most cost-effective strategy when the coverage of CD4 testing is lower than 73% and if the cost of ART is reduced by 40%; with resultant ICERs of $320, and $217 per life year gained respectively. Figure 3 highlights results for the variables found to have the most effect on the ICER of Option B+ considering life years gained in HIV-infected mothers.

**Table 5:** Results from sensitivity analyses on input parameters affecting outcomes in HIV-infected mothers; US$ per life year gained (compared to the current practice) and paediatric outcomes; US$ per DALY averted.

Model parameters	Option A	Option B	Option B+
Base case (US$/LYG in HIV-infected mothers)	314.1	337.6	455.3
ARV coverage among HIV-infected pregnant women			
Best case 100%	312.8	333.6	446.7
Worst case 30%	341.2	443.7	751.5
Coverage of CD4 testing			
Best case 90%	314.1	337.6	455.3
Break-even point–73%	320.1	320.4	455.3
Worst case 30%	328.1	305.0	455.3
Cost of Triple-drug regimen (ART)			
Best case $96.8	194.8	188.1	241.1
Break-even point - $ 105.6	217.8	217.0	282.5
Worst case $290	433.3	487.2	669.4
ART coverage in the general population			
Best case 90%	310.9	357.9	519.4
Worst case 49%	314.4	335.6	449.3
Base case (US$/DALY averted- infants)	37.2	69.0	64.3
Background transmission rate used (22%)			
Best case–15%	47.1	83.7	78.0
Worst case-40%	25.4	46.1	43.0
Peripartum transmission rate with Option A (2.7%)			
Best case–1.3%	36.2	68.1	63.5
Worst case–5.2%	41.7	68.1	63.5
Perinatal transmission rate with ART (1.7%)			
Best case–0.7%	37.7	65.3	60.9
Worst case–4.0%	38.9	75.7	70.5
Cost of ART (US$ 193.60)			
Best case-$96.8	30.6	40.5	69.0
Worst case-$ 290.4	45.5	95.8	57.9
Break-even point-$387	52.9	123.4	52.4

## Discussion

We assessed the cost-effectiveness of the 2010 WHO mother-to-child transmission prevention strategies: Options A and B, and Malawi's Option B+. Analyzing both infant infection outcomes and long-term maternal health outcomes allows us to make a distinction between strategies to prevent mother-to-child transmission whose impact improves only child outcomes and those to treat mothers whose impacts improve both survival in HIV-infected mothers and children. Option A is the most cost-effective alternative in our modelling where we have assumed an ideal scenario with universal CD4 screening available and very high rates of ARV coverage for prophylaxis and provision of ART to eligible women. However, in real-world situations such as Malawi, with low access to CD4 testing, and low levels of ART in treatment-eligible women with Option A, the cost-effectiveness favours Option B and B+. Using the WHO commission on Macroeconomics and health criterion for determining the cost-effectiveness of an intervention [41], we found that Option B+ represents a cost-effective strategy not only for preventing new HIV infections among infants, but also for improving the survival of HIV-infected mothers. In a recent programmatic update, WHO has encouraged countries to consider Option B+ [42].

The lower cost-effectiveness ratios (US $38–68 per DALY averted) derived in our analysis when compared to previous studies can be attributed to the current availability of less expensive and more effective regimens for PMTCT as well as improved coverage of services for HIV-infected mothers modelled here. Sweat and colleagues reported higher cost-effectiveness ratios with SD NVP up to US$ 310 per DALY averted, across eight countries in Sub-Saharan Africa [43]. Another study from Tanzania reported an incremental cost-effectiveness ratio of US$162 per DALY averted [44] for HAART compared to single-dose Nevirapine. A more recent study compares WHO option B with short course prophylaxis in Nigeria and reported an ICER of $111 per DALY averted if there was full PMTCT coverage among pregnant women [45].

From an economic perspective, each of the three options analyzed here results in enormous cost savings by averting new paediatric infections; thereby yielding significant returns on investment. For every dollar invested in the PMTCT programmes, between 2.5 and 4 dollars would be obtained in return from averted lifetime pediatric care, equivalent to saving nearly US$ 51 million from each annual birth cohort.

We believe that our study is the first economic evaluation to also assess maternal health outcomes when using ART regimens as part of prevention strategies for mother to child transmission. Integrating HIV services in the context of ‘eliminating new infections among children and keeping their mothers alive’ in Malawi, as the new Global Plan on MTCT elimination recommends [11], results in favourable outcomes for both mothers and infants while still yielding cost effective results. Under ideal settings where most women in need of ART for their own health and HIV-infected pregnant women who need ARV prophylaxis for PMTCT receive adequate drugs and clinical monitoring, the three options, A, B and B+ included in our analysis, when compared directly with the base case will result in incremental cost-effectiveness ratios that are less than three times the 2010 GDP per capita in Malawi of US$ 310 [41] and can be considered feasible policy options. After ten years of treatment, although Option B+ has significantly higher costs, the wider and earlier use of ART results in greater direct benefits, keeping the most women alive for the longest time, and yielding an incremental cost-effectiveness ratio of US $ 455 per life year gained over the current practice. In the Malawi context, where access to CD4 count and therefore targeted initiation of HIV-infected pregnant women on ART based upon CD4 results is unlikely to be successful given current limitations in CD4 test access, this approach represents a cost-effective policy option to prevent a child from being infected, improve HIV-free survival of infants, save mother's lives and reduce orphanhood.

Increasing access to treatment among pregnant women has several advantages. From a therapeutic point of view, it would reduce morbidity and mortality among HIV infected women. Treatment guidelines in North America and Europe recommend treatment initiation with higher CD4 counts [46] than those recommended for resource-limited settings. Recent results from the HPTN 052 study show that ART is 96% effective in preventing transmission to an uninfected sexual partner in discordant couples where the index case has CD4 counts between 350 and 550 cells/ µL [47] and ART during pregnancy and after may serve as a preventive strategy among discordant couples and to prevent sexual transmission more generally. Future modelling studies are needed to address the potential benefit of Option B+ as a strategy of early start of ART for prevention of new infections in the general population. Early ART is also known to reduce the risk of developing tuberculosis, the leading cause of mortality in HIV infected individuals, which increases with declining CD4 counts below 500cells/ µL [48]. Finally, evidence shows that women on ART before pregnancy can have lower mother-to-child transmission rates compared to those initiating ARV prophylaxis during pregnancy, as low as 0.5% during pregnancy [49] as some transmission can occur before the timing of prophylaxis protocols, and as women may often enter into antenatal care, and hence PMTCT late in pregnancy. Therefore, for countries like Malawi, which has a high fertility rate of 5.5 children per woman, the long-term effect of continuous ART would result in many more infections averted during subsequent pregnancies and improve the cost-effectiveness of Option B+.

As in any other modelling exercise, there are a number of limitations in relation to our assumptions. The successful implementation of these programmes rests on the capacity of overcoming many obstacles to increase access, especially in rural Malawi and at primary care level. The assumption about scaling programmes from their current levels to high aspiration targets should be taken with reservation. The costs and challenge of achieving such high coverage may be underestimated as the success of these programmes will require intensified community participation, human resources and the infrastructure necessary for service delivery. We made every effort to make explicit all the assumptions in our model, which we hope will be timely and critical to inform policy options. One simplifying assumption we made was that women would continue to receive the first-line ART regimen; this does not account for the realities that women face in terms of stigma, adherence and treatment failures requiring the switching of women to more expensive second- or third-line regimens. Finally, in the analysis, we have focused on the cost and cost-effectiveness of preventing new paediatric infections and on the health of the mother - we have not modelled the additional benefit and cost-benefit likely to result from the prevention of new adult infections in serodiscordant couples and partners. While we believe that our findings are robust and generalizable for many high burden countries, our results may not be applicable in some settings, particularly those with low prevalence, replacement feeding rather than breastfeeding for HIV-exposed infants and low fertility.

In conclusion, we present an economic analysis for the Malawi approach that places PMTCT within a continuum of care that integrates a comprehensive range of interventions, to make an impact on maternal, newborn and child health outcomes [10], [50]. Our analysis suggests that Option B+ is a cost-effective strategy to integrate HIV prevention and treatment efforts towards achieving Millennium Development Goals 4, 5 & 6 [51] and ensuring universal access to ART. Careful monitoring of the Malawi approach is needed to assess the programmatic implications and will be critical for contributing to the evidence base for future global guidelines revisions. However, from an economic point of view, while this strategy will certainly require additional short-term financial resources, it has the potential to save significant societal resources in the long term.
